# 2,3,6,3′,4′-Penta-*O*-acetyl-4,1′,6′-tri­chloro-4,1′,6′-tride­oxy­sucrose

**DOI:** 10.1107/S1600536811032120

**Published:** 2011-08-11

**Authors:** Fu-Zhong Wu, Ping Zhang

**Affiliations:** a East China University of Science and Technology, 200237 Shanghai, People’s Republic of China

## Abstract

In the title compound, C_22_H_29_Cl_3_O_13_, the glucopyran ring exists in the chair conformation while the glucofuran ring adopts an envelope conformation. Intra­molecular C—H⋯O hydrogen bonds occur. In the crystal, adjacent mol­ecules are linked by weak inter­molecular C—H⋯O hydrogen bonds.

## Related literature

For general background to sucralose (4,1′,6′-trichloro-4,1′,6′-tride­oxy-*galacto*-sucrose), see: John *et al.* (2000 ▶); Khan (1972 ▶); Mclean (2000 ▶). For details of the synthesis, see: Kille *et al.* (2000 ▶); Wu *et al.* (2010 ▶).
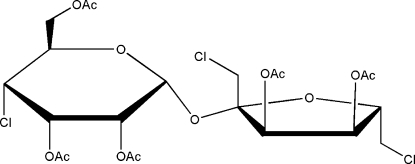


## Experimental

### 

#### Crystal data

C_22_H_29_Cl_3_O_13_
*M* *_r_* = 607.80
Orthorhombic, 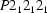

*a* = 8.9813 (6) Å
*b* = 15.5062 (10) Å
*c* = 19.9737 (13) Å
*V* = 2781.7 (3) Å^3^
*Z* = 4
Mo *K*α radiation
μ = 0.39 mm^−1^
*T* = 293 K
0.37 × 0.31 × 0.21 mm

#### Data collection

Bruker SMART CCD area-detector diffractometer
Absorption correction: multi-scan (*SADABS*; Bruker, 2002 ▶) *T*_min_ = 0.777, *T*_max_ = 1.000
15159 measured reflections
5463 independent reflections
5081 reflections with *I* > 2σ(*I*)
*R*_int_ = 0.021

#### Refinement

*R*[*F* ^2^ > 2σ(*F* ^2^)] = 0.037
*wR*(*F* ^2^) = 0.098
*S* = 1.06
5463 reflections
348 parameters
H-atom parameters constrained
Δρ_max_ = 0.32 e Å^−3^
Δρ_min_ = −0.19 e Å^−3^
Absolute structure: Flack (1983 ▶), 2367 Friedel pairs
Flack parameter: 0.00 (5)

### 

Data collection: *SMART* (Bruker, 2002 ▶); cell refinement: *SAINT* (Bruker, 2002 ▶); data reduction: *SAINT*; program(s) used to solve structure: *SHELXTL* (Sheldrick, 2008 ▶); program(s) used to refine structure: *SHELXTL*; molecular graphics: *SHELXTL*; software used to prepare material for publication: *SHELXTL*.

## Supplementary Material

Crystal structure: contains datablock(s) I, global. DOI: 10.1107/S1600536811032120/xu5274sup1.cif

Structure factors: contains datablock(s) I. DOI: 10.1107/S1600536811032120/xu5274Isup2.hkl

Additional supplementary materials:  crystallographic information; 3D view; checkCIF report

## Figures and Tables

**Table 1:** Hydrogen-bond geometry (Å, °)

*D*—H⋯*A*	*D*—H	H⋯*A*	*D*⋯*A*	*D*—H⋯*A*
C12—H12*B*⋯O13	0.96	2.51	3.304 (3)	139
C16—H16*B*⋯O11^i^	0.96	2.57	3.300 (4)	133
C19—H19*B*⋯O3^ii^	0.96	2.40	3.351 (4)	172
C21—H21*C*⋯O3^iii^	0.96	2.44	3.396 (4)	174
C22—H22*A*⋯O1	0.97	2.49	3.321 (3)	143

Symmetry codes: (i) 
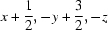
; (ii) 
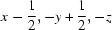
; (iii) 
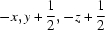
.

**Fig. 1.**
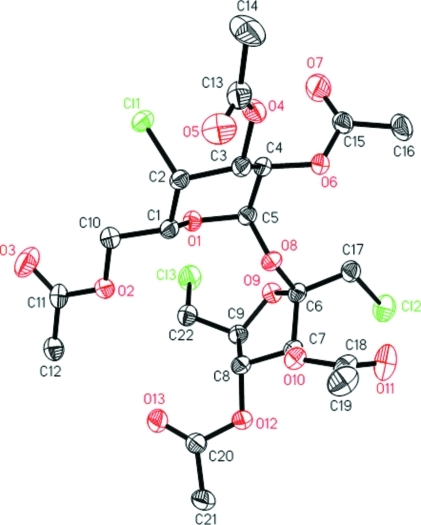
A view of the molecular structure showing ellipsoids at the 30% probability level. Hydrogen atoms have been omitted for clarity.

**Fig. 2.**
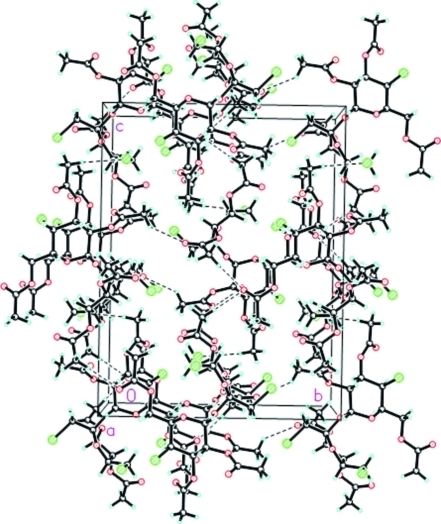
Packing diagram view along the a axis.

## supplementary crystallographic information

### Comment

The well known sucralose (4,1',6'-Trichloro-4,1',6'–trideoxy-*galacto*-sucrose) is a low calorie sweetener made from suger and tastes similar to sugar. It is about 600 times sweeter than suger. Sucralose can be safely consumed and used wherein there is a need to avoid use of sugar. More particularly it is very useful for preparing food, beverages and nutritional product wherein the use of sugar needs to be avoided. The sucralose is used in foods sweetening beverage and nutritional products ingredient world wide. Sucralose is a high-sensitivity artificial sweetener (John *et al.*, 2000; Khan, 1972; Mclean *et al.*, 2000), and many convertional methods of producing sucralose have already been reported (Kille *et al.*, 2000). The title compound (4,1',6'-Trichloro-4,1',6'-trideoxy-2,3,6,3',4'-sucrose pentaacetate), as the key intermediate of sucralose, was obtained by ourselves (Wu *et al.*, 2010). Herein, we report the synthesis, characterization and crystal structure of the title compound.

The compound crystallizes in the orthorhombic space group *P*2_1_2_1_2_1_, with one molecule in the asymmetric unit. The molecule structure consists of a glucopyran ring and a glucofuran ring (Fig. 1). The glucopyran ring exists in the form of chair, while the glucofuran ring adopts envelope conformation. The two rings attach to one oxygen atom by equation bonds. Even though non-classical hydrogen bonds observed in the crystal structure, two kind of weak intermolecular C—H···O and C—H···Cl hydrogen bonds play an important role in the formation of a three-dimensional suparmolecular architecture (Fig. 2).

### Experimental

2,3,6,3',4'-Penta-*O*-acetylsucrose (4.5 mmol) and thionyl chloride (5.0 mmol) in toluene (10 ml) were refluxed for 4 h. Then solvent was removed on a vacuum rotary evaporator. Crude product (2.84 g, 90% yield) was recrystallized from EtOH to give crystals suitable for single-crystal X-ray diffraction (yield 85%).

### Refinement

All H atoms were placed in geometrically idealized positions with C—H = 0.98–0.96 Å and refined in riding mode with U_iso_(H) = 1.5U_eq_(C) for methyl H atoms and 1.2U_eq_(C) for the others.

### Crystal data

**Table:**

C_22_H_29_Cl_3_O_13_	*F*(000) = 1264
*M**_r_* = 607.80	*D*_x_ = 1.451 Mg m^−^^3^
Orthorhombic, *P*2_1_2_1_2_1_	Mo *K*α radiation, λ = 0.71073 Å
Hall symbol: P 2ac 2ab	Cell parameters from 6865 reflections
*a* = 8.9813 (6) Å	θ = 2.4–25.6°
*b* = 15.5062 (10) Å	µ = 0.39 mm^−^^1^
*c* = 19.9737 (13) Å	*T* = 293 K
*V* = 2781.7 (3) Å^3^	Prism, colourless
*Z* = 4	0.37 × 0.31 × 0.21 mm

### Data collection

**Table:**

Bruker SMART CCD area-detector diffractometer	5463 independent reflections
Radiation source: fine-focus sealed tube	5081 reflections with *I* > 2σ(*I*)
graphite	*R*_int_ = 0.021
φ and ω scans	θ_max_ = 26.0°, θ_min_ = 2.0°
Absorption correction: multi-scan (*SADABS*; Bruker, 2002)	*h* = −10→11
*T*_min_ = 0.777, *T*_max_ = 1.000	*k* = −19→12
15159 measured reflections	*l* = −24→24

### Refinement

**Table:**

Refinement on *F*^2^	Secondary atom site location: difference Fourier map
Least-squares matrix: full	Hydrogen site location: inferred from neighbouring sites
*R*[*F*^2^ > 2σ(*F*^2^)] = 0.037	H-atom parameters constrained
*wR*(*F*^2^) = 0.098	*w* = 1/[σ^2^(*F*_o_^2^) + (0.0561*P*)^2^ + 0.3705*P*] where *P* = (*F*_o_^2^ + 2*F*_c_^2^)/3
*S* = 1.06	(Δ/σ)_max_ = 0.007
5463 reflections	Δρ_max_ = 0.32 e Å^−^^3^
348 parameters	Δρ_min_ = −0.19 e Å^−^^3^
0 restraints	Absolute structure: Flack (1983), 2367 Friedel pairs
Primary atom site location: structure-invariant direct methods	Flack parameter: 0.00 (5)

### Special details

**Table:**

Experimental. 1H NMR (400 MHz, DMSO-d6): σ 2.03 (3*H*, s, COCH3), 2.05 (3*H*, s, COCH3), 2.06 (6*H*, s, COCH3), 2.07 (3*H*, s, COCH3), 3.82–3.96 (4*H*, m, 2ClCH2), 4.16 (2*H*, d, J = 5.6 Hz, CH2OAc), 4.26–4.31 (1*H*, m), 4.55 (1*H*, t, J = 5.8 Hz), 4.85 (1*H*, dd, J = 1.0, 3.4 Hz), 5.15 (1*H*, dd, J = 3.6, 8.1 Hz), 5.34–5.40 (2*H*, m), 5.64 (1*H*, d, J = 7.2 Hz), 5.68 (1*H*, d, J = 3.6 Hz).
Geometry. All e.s.d.'s (except the e.s.d. in the dihedral angle between two l.s. planes) are estimated using the full covariance matrix. The cell e.s.d.'s are taken into account individually in the estimation of e.s.d.'s in distances, angles and torsion angles; correlations between e.s.d.'s in cell parameters are only used when they are defined by crystal symmetry. An approximate (isotropic) treatment of cell e.s.d.'s is used for estimating e.s.d.'s involving l.s. planes.
Refinement. Refinement of *F*^2^ against ALL reflections. The weighted *R*-factor *wR* and goodness of fit *S* are based on *F*^2^, conventional *R*-factors *R* are based on *F*, with *F* set to zero for negative *F*^2^. The threshold expression of *F*^2^ > σ(*F*^2^) is used only for calculating *R*-factors(gt) *etc*. and is not relevant to the choice of reflections for refinement. *R*-factors based on *F*^2^ are statistically about twice as large as those based on *F*, and *R*- factors based on ALL data will be even larger.

### Fractional atomic coordinates and isotropic or equivalent isotropic displacement parameters (Å^2^)

**Table:**

	*x*	*y*	*z*	*U*_iso_*/*U*_eq_	
Cl1	0.18975 (7)	0.24388 (4)	−0.12651 (3)	0.05417 (15)	
Cl2	0.09410 (10)	0.70991 (4)	0.09992 (4)	0.0764 (2)	
Cl3	0.38581 (8)	0.40249 (5)	0.17639 (5)	0.0757 (2)	
O1	0.16446 (17)	0.35433 (9)	0.00841 (7)	0.0428 (3)	
O2	0.0328 (2)	0.21775 (11)	0.08279 (9)	0.0582 (5)	
O3	0.1521 (3)	0.09365 (15)	0.09253 (11)	0.0966 (9)	
O4	0.0716 (2)	0.39925 (11)	−0.18950 (8)	0.0562 (4)	
O5	−0.1314 (2)	0.32350 (16)	−0.21651 (11)	0.0778 (6)	
O6	0.14003 (19)	0.53711 (9)	−0.10439 (8)	0.0482 (4)	
O7	0.3616 (3)	0.54004 (15)	−0.15533 (15)	0.0985 (8)	
O8	0.02654 (16)	0.48044 (9)	0.01512 (7)	0.0415 (3)	
O9	0.15910 (17)	0.51900 (10)	0.11205 (7)	0.0447 (3)	
O10	−0.23093 (18)	0.51482 (10)	0.07552 (8)	0.0484 (4)	
O11	−0.2683 (3)	0.65461 (13)	0.05707 (14)	0.0862 (7)	
O12	−0.1484 (2)	0.47132 (10)	0.21759 (8)	0.0522 (4)	
O13	−0.1041 (3)	0.33524 (11)	0.24732 (9)	0.0669 (5)	
C1	0.0421 (2)	0.30399 (14)	−0.01580 (11)	0.0421 (5)	
H1	−0.0509	0.3286	0.0013	0.050*	
C2	0.0366 (2)	0.30306 (14)	−0.09186 (11)	0.0403 (4)	
H2	−0.0562	0.2750	−0.1058	0.048*	
C3	0.0374 (2)	0.39501 (13)	−0.11934 (10)	0.0417 (5)	
H3	−0.0595	0.4220	−0.1111	0.050*	
C4	0.1589 (2)	0.44793 (13)	−0.08635 (11)	0.0403 (5)	
H4	0.2559	0.4280	−0.1026	0.048*	
C5	0.1564 (2)	0.44207 (13)	−0.01042 (11)	0.0398 (5)	
H5	0.2433	0.4725	0.0075	0.048*	
C6	0.0430 (3)	0.54111 (14)	0.06840 (10)	0.0410 (5)	
C7	−0.0980 (2)	0.53377 (14)	0.11150 (10)	0.0424 (5)	
H7	−0.1114	0.5867	0.1376	0.051*	
C8	−0.0613 (3)	0.46004 (15)	0.15774 (11)	0.0445 (5)	
H8	−0.0838	0.4046	0.1365	0.053*	
C9	0.1065 (3)	0.47010 (14)	0.16894 (10)	0.0449 (5)	
H9	0.1240	0.5030	0.2101	0.054*	
C10	0.0605 (3)	0.21462 (15)	0.01212 (13)	0.0539 (6)	
H10A	−0.0091	0.1755	−0.0092	0.065*	
H10B	0.1607	0.1939	0.0037	0.065*	
C11	0.0786 (3)	0.14943 (14)	0.11772 (13)	0.0513 (6)	
C12	0.0301 (3)	0.15198 (16)	0.18838 (13)	0.0567 (6)	
H12A	−0.0737	0.1375	0.1911	0.085*	
H12B	0.0455	0.2089	0.2061	0.085*	
H12C	0.0870	0.1112	0.2139	0.085*	
C13	−0.0184 (4)	0.35766 (17)	−0.23298 (13)	0.0618 (7)	
C14	0.0489 (6)	0.3574 (3)	−0.30054 (15)	0.1107 (16)	
H14A	0.1269	0.3151	−0.3023	0.166*	
H14B	0.0895	0.4133	−0.3101	0.166*	
H14C	−0.0259	0.3435	−0.3331	0.166*	
C15	0.2519 (3)	0.57680 (15)	−0.13722 (12)	0.0542 (6)	
C16	0.2162 (4)	0.66902 (16)	−0.14929 (15)	0.0766 (9)	
H16A	0.3024	0.6978	−0.1668	0.115*	
H16B	0.1870	0.6957	−0.1079	0.115*	
H16C	0.1360	0.6732	−0.1809	0.115*	
C17	0.0780 (3)	0.62800 (15)	0.03785 (12)	0.0502 (5)	
H17A	−0.0003	0.6435	0.0066	0.060*	
H17B	0.1706	0.6242	0.0130	0.060*	
C18	−0.3104 (3)	0.58228 (18)	0.05202 (13)	0.0558 (6)	
C19	−0.4519 (3)	0.5537 (2)	0.02097 (17)	0.0764 (9)	
H19A	−0.5134	0.5267	0.0543	0.115*	
H19B	−0.4310	0.5131	−0.0141	0.115*	
H19C	−0.5031	0.6026	0.0026	0.115*	
C20	−0.1637 (3)	0.40117 (15)	0.25818 (12)	0.0497 (5)	
C21	−0.2646 (4)	0.42122 (19)	0.31566 (15)	0.0718 (8)	
H21A	−0.2709	0.3720	0.3446	0.108*	
H21B	−0.3620	0.4351	0.2991	0.108*	
H21C	−0.2257	0.4694	0.3402	0.108*	
C22	0.1891 (3)	0.38625 (16)	0.17245 (13)	0.0522 (6)	
H22A	0.1653	0.3519	0.1333	0.063*	
H22B	0.1569	0.3545	0.2117	0.063*	

### Atomic displacement parameters (Å^2^)

**Table:**

	*U*^11^	*U*^22^	*U*^33^	*U*^12^	*U*^13^	*U*^23^
Cl1	0.0536 (3)	0.0469 (3)	0.0621 (3)	0.0062 (3)	0.0132 (3)	−0.0079 (3)
Cl2	0.0967 (6)	0.0442 (3)	0.0881 (5)	−0.0107 (4)	0.0013 (4)	−0.0182 (3)
Cl3	0.0544 (4)	0.0727 (5)	0.1000 (6)	0.0109 (3)	−0.0020 (4)	0.0109 (4)
O1	0.0459 (8)	0.0387 (7)	0.0437 (8)	0.0032 (6)	−0.0028 (6)	0.0014 (6)
O2	0.0691 (11)	0.0485 (9)	0.0569 (10)	0.0143 (9)	0.0188 (9)	0.0138 (8)
O3	0.156 (2)	0.0704 (13)	0.0633 (12)	0.0511 (16)	−0.0096 (14)	−0.0051 (10)
O4	0.0768 (12)	0.0511 (9)	0.0408 (8)	−0.0128 (9)	−0.0033 (8)	−0.0013 (7)
O5	0.0640 (12)	0.0976 (16)	0.0719 (13)	−0.0123 (12)	−0.0156 (10)	−0.0165 (11)
O6	0.0612 (9)	0.0342 (7)	0.0492 (8)	0.0014 (7)	0.0071 (7)	0.0001 (6)
O7	0.0832 (16)	0.0680 (14)	0.144 (2)	−0.0071 (12)	0.0512 (16)	0.0181 (14)
O8	0.0402 (7)	0.0437 (8)	0.0405 (7)	0.0026 (7)	−0.0018 (6)	−0.0080 (6)
O9	0.0444 (8)	0.0460 (8)	0.0436 (8)	−0.0028 (7)	−0.0055 (6)	−0.0007 (6)
O10	0.0451 (9)	0.0479 (9)	0.0524 (8)	0.0026 (7)	−0.0029 (7)	−0.0070 (7)
O11	0.0912 (16)	0.0523 (11)	0.1152 (18)	0.0098 (11)	−0.0340 (14)	−0.0051 (12)
O12	0.0643 (10)	0.0472 (9)	0.0452 (8)	0.0014 (8)	0.0120 (7)	−0.0034 (7)
O13	0.0935 (14)	0.0459 (10)	0.0611 (11)	0.0005 (10)	0.0128 (10)	−0.0030 (8)
C1	0.0393 (11)	0.0385 (11)	0.0485 (11)	0.0020 (9)	0.0056 (9)	−0.0016 (9)
C2	0.0357 (10)	0.0373 (11)	0.0480 (11)	−0.0008 (9)	0.0042 (9)	−0.0054 (9)
C3	0.0472 (11)	0.0386 (11)	0.0392 (11)	0.0020 (9)	−0.0001 (9)	−0.0019 (9)
C4	0.0444 (12)	0.0336 (10)	0.0430 (11)	0.0009 (9)	0.0037 (9)	−0.0019 (8)
C5	0.0358 (11)	0.0398 (10)	0.0439 (11)	0.0010 (8)	−0.0004 (8)	−0.0029 (8)
C6	0.0457 (11)	0.0369 (10)	0.0405 (10)	0.0012 (9)	−0.0032 (9)	−0.0063 (9)
C7	0.0458 (11)	0.0409 (11)	0.0406 (11)	0.0009 (9)	−0.0020 (9)	−0.0077 (9)
C8	0.0501 (12)	0.0417 (11)	0.0418 (11)	0.0013 (10)	0.0045 (9)	−0.0069 (9)
C9	0.0556 (13)	0.0433 (11)	0.0357 (10)	−0.0005 (10)	−0.0020 (9)	−0.0058 (9)
C10	0.0616 (15)	0.0431 (12)	0.0571 (14)	0.0010 (11)	0.0108 (12)	0.0034 (10)
C11	0.0585 (14)	0.0362 (11)	0.0591 (13)	0.0015 (10)	−0.0091 (11)	0.0018 (10)
C12	0.0630 (15)	0.0497 (14)	0.0573 (14)	0.0026 (12)	−0.0040 (12)	0.0086 (11)
C13	0.084 (2)	0.0514 (14)	0.0502 (13)	−0.0030 (14)	−0.0166 (13)	−0.0065 (11)
C14	0.188 (5)	0.098 (3)	0.0462 (15)	−0.057 (3)	−0.001 (2)	−0.0106 (16)
C15	0.0730 (17)	0.0422 (12)	0.0473 (13)	−0.0118 (12)	0.0048 (12)	−0.0021 (10)
C16	0.123 (3)	0.0427 (13)	0.0645 (17)	−0.0157 (17)	0.0068 (18)	0.0037 (12)
C17	0.0589 (14)	0.0414 (12)	0.0503 (12)	−0.0015 (11)	0.0000 (10)	−0.0030 (10)
C18	0.0555 (13)	0.0609 (16)	0.0511 (13)	0.0152 (12)	−0.0035 (11)	−0.0094 (12)
C19	0.0586 (16)	0.093 (2)	0.0774 (19)	0.0142 (16)	−0.0169 (15)	−0.0131 (17)
C20	0.0597 (14)	0.0471 (13)	0.0422 (12)	−0.0086 (11)	0.0023 (10)	−0.0077 (10)
C21	0.088 (2)	0.0648 (17)	0.0623 (16)	−0.0054 (16)	0.0263 (15)	−0.0004 (14)
C22	0.0555 (13)	0.0489 (13)	0.0523 (13)	0.0033 (11)	−0.0045 (11)	−0.0011 (10)

### Geometric parameters (Å, °)

**Table:**

Cl1—C2	1.793 (2)	C6—C17	1.512 (3)
Cl2—C17	1.781 (2)	C6—C7	1.536 (3)
Cl3—C22	1.786 (3)	C7—C8	1.506 (3)
O1—C5	1.414 (3)	C7—H7	0.9800
O1—C1	1.432 (3)	C8—C9	1.532 (3)
O2—C11	1.333 (3)	C8—H8	0.9800
O2—C10	1.434 (3)	C9—C22	1.498 (3)
O3—C11	1.199 (3)	C9—H9	0.9800
O4—C13	1.351 (3)	C10—H10A	0.9700
O4—C3	1.436 (3)	C10—H10B	0.9700
O5—C13	1.191 (4)	C11—C12	1.478 (4)
O6—C15	1.348 (3)	C12—H12A	0.9600
O6—C4	1.439 (2)	C12—H12B	0.9600
O7—C15	1.195 (3)	C12—H12C	0.9600
O8—C5	1.405 (3)	C13—C14	1.479 (5)
O8—C6	1.428 (2)	C14—H14A	0.9600
O9—C6	1.402 (3)	C14—H14B	0.9600
O9—C9	1.445 (3)	C14—H14C	0.9600
O10—C18	1.350 (3)	C15—C16	1.485 (4)
O10—C7	1.424 (3)	C16—H16A	0.9600
O11—C18	1.188 (3)	C16—H16B	0.9600
O12—C20	1.364 (3)	C16—H16C	0.9600
O12—C8	1.439 (3)	C17—H17A	0.9700
O13—C20	1.174 (3)	C17—H17B	0.9700
C1—C10	1.503 (3)	C18—C19	1.483 (4)
C1—C2	1.520 (3)	C19—H19A	0.9600
C1—H1	0.9800	C19—H19B	0.9600
C2—C3	1.528 (3)	C19—H19C	0.9600
C2—H2	0.9800	C20—C21	1.495 (4)
C3—C4	1.516 (3)	C21—H21A	0.9600
C3—H3	0.9800	C21—H21B	0.9600
C4—C5	1.519 (3)	C21—H21C	0.9600
C4—H4	0.9800	C22—H22A	0.9700
C5—H5	0.9800	C22—H22B	0.9700
			
C5—O1—C1	113.30 (16)	O2—C10—H10A	110.0
C11—O2—C10	115.76 (19)	C1—C10—H10A	110.0
C13—O4—C3	118.6 (2)	O2—C10—H10B	110.0
C15—O6—C4	118.21 (19)	C1—C10—H10B	110.0
C5—O8—C6	117.60 (16)	H10A—C10—H10B	108.4
C6—O9—C9	111.99 (16)	O3—C11—O2	121.6 (2)
C18—O10—C7	117.29 (19)	O3—C11—C12	125.6 (2)
C20—O12—C8	116.86 (18)	O2—C11—C12	112.8 (2)
O1—C1—C10	107.04 (18)	C11—C12—H12A	109.5
O1—C1—C2	111.57 (17)	C11—C12—H12B	109.5
C10—C1—C2	111.45 (18)	H12A—C12—H12B	109.5
O1—C1—H1	108.9	C11—C12—H12C	109.5
C10—C1—H1	108.9	H12A—C12—H12C	109.5
C2—C1—H1	108.9	H12B—C12—H12C	109.5
C1—C2—C3	110.49 (17)	O5—C13—O4	123.0 (3)
C1—C2—Cl1	111.46 (16)	O5—C13—C14	126.8 (3)
C3—C2—Cl1	109.58 (15)	O4—C13—C14	110.1 (3)
C1—C2—H2	108.4	C13—C14—H14A	109.5
C3—C2—H2	108.4	C13—C14—H14B	109.5
Cl1—C2—H2	108.4	H14A—C14—H14B	109.5
O4—C3—C4	104.23 (18)	C13—C14—H14C	109.5
O4—C3—C2	113.23 (17)	H14A—C14—H14C	109.5
C4—C3—C2	110.64 (18)	H14B—C14—H14C	109.5
O4—C3—H3	109.5	O7—C15—O6	123.0 (2)
C4—C3—H3	109.5	O7—C15—C16	126.0 (3)
C2—C3—H3	109.5	O6—C15—C16	111.0 (3)
O6—C4—C3	109.05 (18)	C15—C16—H16A	109.5
O6—C4—C5	107.80 (16)	C15—C16—H16B	109.5
C3—C4—C5	113.00 (18)	H16A—C16—H16B	109.5
O6—C4—H4	109.0	C15—C16—H16C	109.5
C3—C4—H4	109.0	H16A—C16—H16C	109.5
C5—C4—H4	109.0	H16B—C16—H16C	109.5
O8—C5—O1	110.70 (17)	C6—C17—Cl2	111.80 (16)
O8—C5—C4	110.45 (17)	C6—C17—H17A	109.3
O1—C5—C4	108.81 (17)	Cl2—C17—H17A	109.3
O8—C5—H5	108.9	C6—C17—H17B	109.3
O1—C5—H5	108.9	Cl2—C17—H17B	109.3
C4—C5—H5	108.9	H17A—C17—H17B	107.9
O9—C6—O8	112.31 (17)	O11—C18—O10	122.3 (3)
O9—C6—C17	108.34 (18)	O11—C18—C19	126.2 (3)
O8—C6—C17	107.93 (18)	O10—C18—C19	111.5 (2)
O9—C6—C7	104.27 (16)	C18—C19—H19A	109.5
O8—C6—C7	106.46 (17)	C18—C19—H19B	109.5
C17—C6—C7	117.62 (19)	H19A—C19—H19B	109.5
O10—C7—C8	109.63 (18)	C18—C19—H19C	109.5
O10—C7—C6	115.08 (17)	H19A—C19—H19C	109.5
C8—C7—C6	102.71 (17)	H19B—C19—H19C	109.5
O10—C7—H7	109.7	O13—C20—O12	122.6 (2)
C8—C7—H7	109.7	O13—C20—C21	126.8 (2)
C6—C7—H7	109.7	O12—C20—C21	110.6 (2)
O12—C8—C7	107.36 (17)	C20—C21—H21A	109.5
O12—C8—C9	113.64 (18)	C20—C21—H21B	109.5
C7—C8—C9	103.13 (18)	H21A—C21—H21B	109.5
O12—C8—H8	110.8	C20—C21—H21C	109.5
C7—C8—H8	110.8	H21A—C21—H21C	109.5
C9—C8—H8	110.8	H21B—C21—H21C	109.5
O9—C9—C22	109.29 (18)	C9—C22—Cl3	111.67 (17)
O9—C9—C8	105.08 (17)	C9—C22—H22A	109.3
C22—C9—C8	113.9 (2)	Cl3—C22—H22A	109.3
O9—C9—H9	109.5	C9—C22—H22B	109.3
C22—C9—H9	109.5	Cl3—C22—H22B	109.3
C8—C9—H9	109.5	H22A—C22—H22B	107.9
O2—C10—C1	108.36 (18)		
			
C5—O1—C1—C10	−176.05 (17)	O8—C6—C7—O10	−34.1 (2)
C5—O1—C1—C2	61.8 (2)	C17—C6—C7—O10	87.0 (2)
O1—C1—C2—C3	−53.4 (2)	O9—C6—C7—C8	−34.0 (2)
C10—C1—C2—C3	−172.96 (19)	O8—C6—C7—C8	84.92 (19)
O1—C1—C2—Cl1	68.7 (2)	C17—C6—C7—C8	−153.96 (19)
C10—C1—C2—Cl1	−50.9 (2)	C20—O12—C8—C7	162.76 (19)
C13—O4—C3—C4	−179.9 (2)	C20—O12—C8—C9	−83.9 (2)
C13—O4—C3—C2	59.8 (3)	O10—C7—C8—O12	−82.9 (2)
C1—C2—C3—O4	164.59 (19)	C6—C7—C8—O12	154.33 (17)
Cl1—C2—C3—O4	41.4 (2)	O10—C7—C8—C9	156.84 (16)
C1—C2—C3—C4	48.0 (2)	C6—C7—C8—C9	34.0 (2)
Cl1—C2—C3—C4	−75.18 (19)	C6—O9—C9—C22	123.71 (19)
C15—O6—C4—C3	−120.1 (2)	C6—O9—C9—C8	1.1 (2)
C15—O6—C4—C5	116.9 (2)	O12—C8—C9—O9	−138.49 (17)
O4—C3—C4—O6	68.0 (2)	C7—C8—C9—O9	−22.6 (2)
C2—C3—C4—O6	−169.99 (16)	O12—C8—C9—C22	101.9 (2)
O4—C3—C4—C5	−172.15 (17)	C7—C8—C9—C22	−142.19 (19)
C2—C3—C4—C5	−50.1 (2)	C11—O2—C10—C1	−164.5 (2)
C6—O8—C5—O1	108.77 (19)	O1—C1—C10—O2	70.0 (2)
C6—O8—C5—C4	−130.65 (18)	C2—C1—C10—O2	−167.7 (2)
C1—O1—C5—O8	60.5 (2)	C10—O2—C11—O3	7.7 (4)
C1—O1—C5—C4	−61.1 (2)	C10—O2—C11—C12	−172.8 (2)
O6—C4—C5—O8	54.3 (2)	C3—O4—C13—O5	6.3 (4)
C3—C4—C5—O8	−66.3 (2)	C3—O4—C13—C14	−170.4 (3)
O6—C4—C5—O1	176.01 (16)	C4—O6—C15—O7	4.5 (4)
C3—C4—C5—O1	55.4 (2)	C4—O6—C15—C16	−177.7 (2)
C9—O9—C6—O8	−94.3 (2)	O9—C6—C17—Cl2	−60.4 (2)
C9—O9—C6—C17	146.60 (17)	O8—C6—C17—Cl2	177.80 (15)
C9—O9—C6—C7	20.6 (2)	C7—C6—C17—Cl2	57.4 (2)
C5—O8—C6—O9	−35.1 (2)	C7—O10—C18—O11	4.5 (4)
C5—O8—C6—C17	84.3 (2)	C7—O10—C18—C19	−175.0 (2)
C5—O8—C6—C7	−148.60 (17)	C8—O12—C20—O13	3.4 (4)
C18—O10—C7—C8	155.65 (19)	C8—O12—C20—C21	−176.3 (2)
C18—O10—C7—C6	−89.2 (2)	O9—C9—C22—Cl3	56.5 (2)
O9—C6—C7—O10	−153.05 (17)	C8—C9—C22—Cl3	173.66 (16)

### Hydrogen-bond geometry (Å, °)

**Table:**

*D*—H···*A*	*D*—H	H···*A*	*D*···*A*	*D*—H···*A*
C12—H12B···O13	0.96	2.51	3.304 (3)	139
C16—H16B···O11^i^	0.96	2.57	3.300 (4)	133
C19—H19B···O3^ii^	0.96	2.40	3.351 (4)	172
C21—H21C···O3^iii^	0.96	2.44	3.396 (4)	174
C22—H22A···O1	0.97	2.49	3.321 (3)	143

Symmetry codes: (i) *x*+1/2, −*y*+3/2, −*z*; (ii) *x*−1/2, −*y*+1/2, −*z*; (iii) −*x*, *y*+1/2, −*z*+1/2.
